# Effects of Field-Map Distortion Correction on Resting State Functional Connectivity MRI

**DOI:** 10.3389/fnins.2017.00656

**Published:** 2017-12-01

**Authors:** Hiroki Togo, Jaroslav Rokicki, Kenji Yoshinaga, Tatsuhiro Hisatsune, Hiroshi Matsuda, Nobuhiko Haga, Takashi Hanakawa

**Affiliations:** ^1^Department of Advanced Neuroimaging, Integrative Brain Imaging Center, National Center of Neurology and Psychiatry, Tokyo, Japan; ^2^Department of Rehabilitation Medicine, Sensory and Motor System Medicine, Graduate School of Medicine, University of Tokyo, Tokyo, Japan; ^3^Japan Society for the Promotion of Science (JSPS), Tokyo, Japan; ^4^Norwegian Centre of Excellence for Mental Disorders Research (NORMENT), KG Jebsen Centre for Psychosis Research, Division of Mental Health and Addiction, Oslo University Hospital, Institute of Clinical Medicine, University of Oslo, Oslo, Norway; ^5^Department of Clinical Neuroimaging, Integrative Brain Imaging Center, National Center of Neurology and Psychiatry, Tokyo, Japan; ^6^Department of Neurology, Graduate School of Medicine, Kyoto University, Kyoto, Japan; ^7^Department of Integrated Biosciences, Graduate School of Frontier Sciences, University of Tokyo, Chiba, Japan

**Keywords:** resting state fMRI, field map, distortion correction, functional connectivity, spectrogram

## Abstract

Magnetic field inhomogeneities cause geometric distortions of echo planar images used for functional magnetic resonance imaging (fMRI). To reduce this problem, distortion correction (DC) with field map is widely used for both task and resting-state fMRI (rs-fMRI). Although DC with field map has been reported to improve the quality of task fMRI, little is known about its effects on rs-fMRI. Here, we tested the influence of field-map DC on rs-fMRI results using two rs-fMRI datasets derived from 40 healthy subjects: one with DC (DC+) and the other without correction (DC−). Independent component analysis followed by the dual regression approach was used for evaluation of resting-state functional connectivity networks (RSN). We also obtained the ratio of low-frequency to high-frequency signal power (0.01–0.1 Hz and above 0.1 Hz, respectively; LFHF ratio) to assess the quality of rs-fMRI signals. For comparison of RSN between DC+ and DC− datasets, the default mode network showed more robust functional connectivity in the DC+ dataset than the DC− dataset. Basal ganglia RSN showed some decreases in functional connectivity primarily in white matter, indicating imperfect registration/normalization without DC. Supplementary seed-based and simulation analyses supported the utility of DC. Furthermore, we found a higher LFHF ratio after field map correction in the anterior cingulate cortex, posterior cingulate cortex, ventral striatum, and cerebellum. In conclusion, field map DC improved detection of functional connectivity derived from low-frequency rs-fMRI signals. We encourage researchers to include a DC step in the preprocessing pipeline of rs-fMRI analysis.

## Introduction

Magnetic resonance images (MRIs) are inherently subject to geometric distortions caused by magnetic field inhomogeneity, and excessive magnetic field inhomogeneity can even result in signal loss (Ojemann et al., 1997). A main source of magnetic inhomogeneity is variability of various tissues' magnetic susceptibility, and geometric distortions are especially pronounced at tissue borders/edges. Susceptibility to magnetic field inhomogeneity depends substantially on MRI sequence, shimming levels, and environmental and experimental factors. Single-shot echo planar imaging (EPI) is one of the most vulnerable MRI sequences to distortion, although it is the fastest common MRI acquisition technique (Mansfield, 1977) and is now used extensively for functional MRI (fMRI). EPI's vulnerability comes from the way in which it fills in the k-space (i.e., the grid of raw data that yields the MRI after Fourier transformation). In single-shot, gradient-echo EPI, a slice's k-space is filled at once after a single excitation following a radiofrequency (RF) pulse; then, rapid gradient switching is used to acquire gradient-echo signals during a free induction decay (FID) period. On the one hand, because this signal is sensitive to magnetic inhomogeneity (i.e., T2^*^-weighted), EPI is useful for fMRI detection of blood oxygenation level-dependent (BOLD) signals coupled with neural/synaptic activity changes in the brain. On the other, phase encoding errors due to magnetic inhomogeneity accumulate during the FID period, resulting in mislocalization of signal sources. Hence, EPI data distortion is pronounced in the phase encoding direction (Jezzard and Balaban, 1995). This characteristic contrasts with other MRI sequences for which phase encoding errors can be compensated (e.g., with a 180° RF pulse) after each RF pulse used to fill the k-space.

A few methods have been proposed to correct for distortion in EPI. One is to acquire EPI in two different encoding directions (Andersson et al., 2003). A more conventional method uses a field map (Jezzard and Balaban, 1995; Jezzard and Clare, 1999; Cusack et al., 2003), which is an image that represents magnetic field intensity across the space. A field map can be measured for each subject's brain, and it provides knowledge about the distribution of the static magnetic field (B0) to correct for geometric distortion in MRI. Distortion correction (DC) has two advantages: it improves registration between functional and structural images, and it reduces the variability of distortion between subjects after spatial normalization to a standard space. Despite its merits, DC is not routinely performed in many types of fMRI studies, especially when the areas of interest are not particularly vulnerable to distortion. Some of the most vulnerable areas in brain MR are the frontal lobe proximal to the paranasal sinuses and temporal lobe proximal to the mastoid air cells and ear canals (Jezzard and Balaban, 1995; Hutton, 2002). Signal loss may also be found in these areas (Devlin et al., 2000; Deichmann et al., 2002).

DC effectively improves the quality of results of task-fMRI with motor tapping and auditory tasks (Cusack et al., 2003). To our knowledge, however, no direct evidence has shown the extent to which DC is effective for resting-state fMRI (rs-fMRI), an emerging fMRI method that examines intrinsic brain activity as slow (typically 0.01–0.1 Hz), spontaneous BOLD signal fluctuations in at-rest fMRI time series. A pioneering study by Biswal et al. (1995) established that over a dozen resting-state networks (RSNs) can be identified as brain regions with correlated temporal patterns of BOLD signals (Fox et al., 2007; Smith et al., 2009). Because of the task-free nature of rs-fMRI, it has many advantages for clinical application over task fMRI and is a powerful tool to provide biomarkers for many neuropsychiatric disorders (Takamura and Hanakawa, 2017).

Here, we hypothesized that DC would increase the useful signal-to-noise ratio for rs-fMRI at the group level, thereby increasing rs-fMRI's detection efficiency for RSNs near the edges of different tissues. To test this hypothesis, we analyzed rs-fMRI datasets with and without DC (DC+ and DC−, respectively) and characterized the areas and the extent to which DC improves rs-fMRI quality. Specifically, we performed independent component analysis followed by dual regression (Filippini et al., 2009) to compare functional connectivity in a set of RSNs of interest between the DC+ and DC− datasets. We also analyzed the quality of rs-fMRI signals using the ratio of low-frequency (0.01–0.1 Hz) to high-frequency (above 0.1 Hz) BOLD signal power, including rs-fMRI signals in which noise components predominated.

## Material and methods

### Participants

A total of 40 healthy older adult participants (age 68.4 ± 5.8 years, 24 male/16 female) were recruited. Each participant gave written informed consent to participate in the study in accordance with the Declaration of Helsinki. The study was approved by the Ethics Committee of the National Center of Neurology and Psychiatry. The exclusion criteria were as follows:

1. Preexisting neuropsychiatric disorders or head injuries.
2. Contraindications to MRI.
3. Mini-mental state examination score below 24 (Folstein et al., 1975).
4. Local brain lesions (e.g., brain tumor or cerebral infarction) incidentally identified on MRI.

### Data acquisition

The rs-fMRI experiments were performed on a 3-T scanner (Siemens, MAGNETOM Verio) at the Integrative Brain Imaging Center, National Center of Neurology and Psychiatry (Tokyo, Japan). All data were acquired using a 32-channel phased array head coil. In the scanner, foam cushions and earplugs were used to limit head motion and reduce scanner noise, respectively. Rs-fMRI scans were acquired using a gradient-echo EPI sequence with repetition time (TR) 2,500 ms, echo time (TE) 30 ms, flip angle 80°, 49 axial slices, slice thickness 3.2 mm (0.8-mm gaps), and a 64 × 64 acquisition matrix, resulting in a 3.3 × 3.3 × 4.0-mm^3^ voxel size. EPI encoding proceeded in the posterior–anterior direction. All subjects underwent a 10-min rs-fMRI scan (240 volumes). They were instructed to remain awake and not to think of anything particular with their eyes open and fixating on a cross hair.

Field map imaging was performed with a double-echo spoiled gradient echo sequence (gre_field_map; TR = 488.0 ms, TE = 4.92/7.38 ms, voxel size: 3.3 × 3.3 × 3.2 (0.8-mm gaps), flip angle 60°) that generated a magnitude image and 2 phase images. The field map image was computed from the 2 phase images. For registration, a whole-brain high-resolution T1-weighted anatomical scan was acquired using a magnetization prepared rapid gradient echo (MP-RAGE) sequence with the following parameters: TR = 1900 ms, TE = 2.52 ms, inversion time (TI) = 900 ms, flip angle = 9 °, field of view = 250 × 250 mm, acquisition matrix = 246 × 256, slice thickness = 1.0 mm without gap, axial slice number = 192, voxel dimension = 1.00 × 0.98 × 0.98, reconstructed as 1.00 × 1.02 × 0.98.

### Data preprocessing

For structural data preprocessing, we removed non-brain tissues and cerebrospinal fluid (CSF) using the Statistical Parametric Mapping software package version 12.

**Figure 1:**
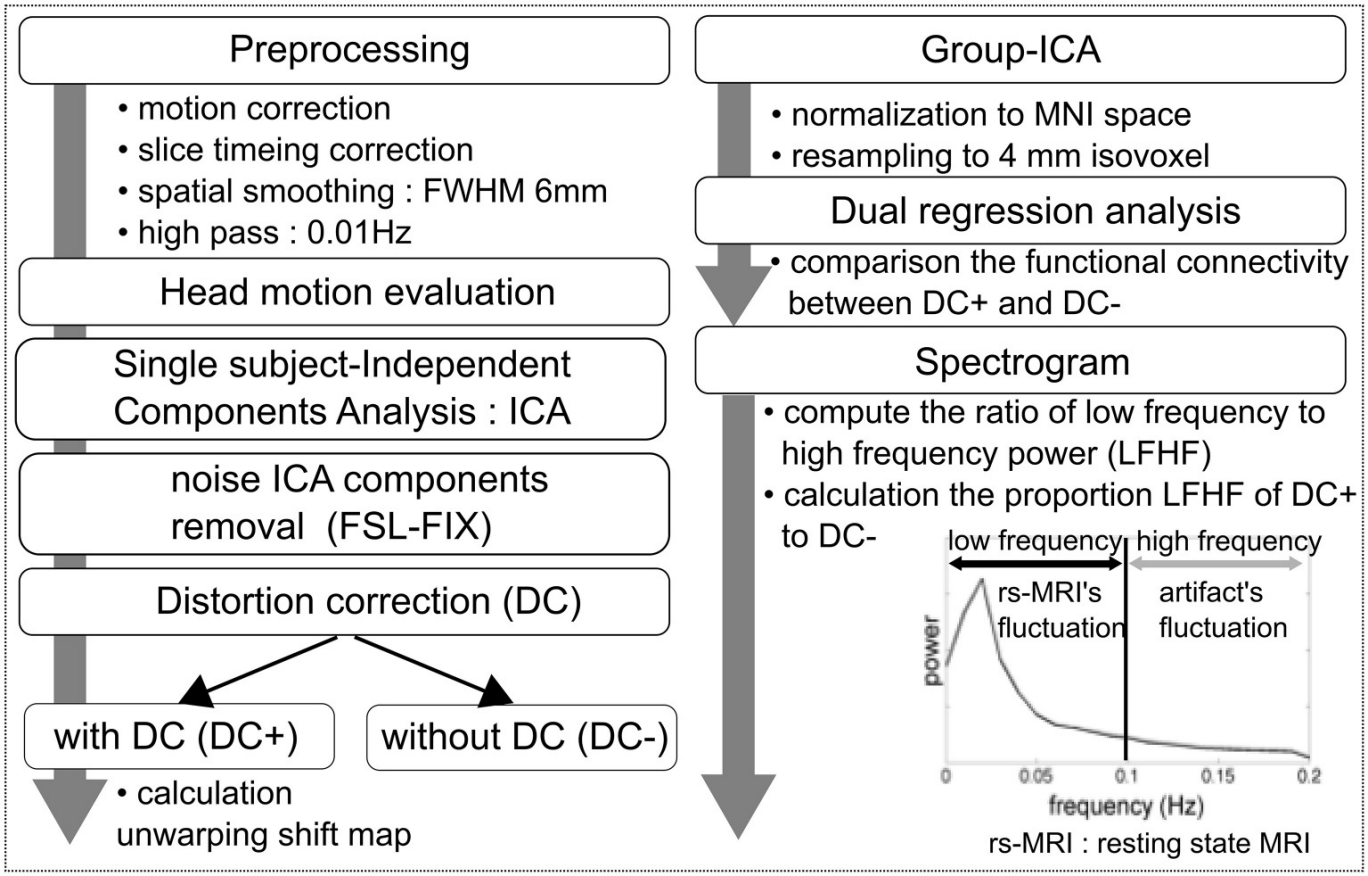
Workflow overview.

Figure 1 shows an overview of the workflow. Rs-fMRI data preprocessing was carried out using FEAT (FMRI Expert Analysis Tool) Version 6.00, which is part of FSL (FMRIB's Software Library, www.fmrib.ox.ac.uk/fsl). Initial preprocessing steps included deleting the first 3 volumes of each fMRI series to allow the magnetic field to reach a steady state, followed by motion correction, spatial smoothing using a 6-mm full-width-at-half-maximum Gaussian kernel, and high-pass temporal filtering with a cutoff frequency of 0.01 Hz. The functional images were coregistered to the high-resolution T1-weighted images using boundary-based registration (Greve and Fischl, 2009).

To assess head motion and noise artifacts, two steps approach was taken. First, we performed a data quality check (especially in terms of head motion) separately for translation and rotation parameters using the following formula:

$$\begin{array}{l} \frac{1}{M-1}\overset{M}{\underset{i=2}{\sum }}\sqrt{|x_{i}-x_{i-1}{|}^{2}+|y_{i}-\;y_{i-1}{|}^{2}+|z_{i}-z_{i-1}{|}^{2}} \end{array}$$

where *M* is the total number of time points, and *x*_*i*_, *y*_*i*_, and *z*_*i*_ are translations or rotations in the three axes at time point *i*, calculated with FEAT in preprocessing step. One subject was excluded from further analysis because of excessive head motion (i.e., translation > 0.3 mm or rotation > 0.3°) (Liu et al., 2008).

Next, single-session independent component analysis (ICA) was performed using Multivariate Exploratory Linear Optimized Decomposition into Independ Components (MELODIC) to decompose the single-subject 4D datasets into sets of spatial and temporal components. Subsequently, to remove noise components from the 4D fMRI data, autoclassification of artifactual ICA spatial components was performed using the FMRIB's ICA-based Xnoiseifier (FIX) (Salimi-Khorshidi et al., 2014). FIX was trained according to the rs-fMRI data from 44 healthy subjects randomly selected from a database (including the participants of the present study) whose images were acquired on the same scanner. Signal vs. noise ICA components were identified by JR and HT using the procedures described by Kelly (Kelly et al., 2010). The identified noise components were regressed out of the data.

The field map correction was applied to the noise-cleaned rs-fMRI data with FMRIB's Utility for Geometrically Unwarping EPIs (FUGUE), part of FSL package. Before the correction procedure, the magnitude images were used to create brain masks, and the phase images were calibrated to units of radians/s. FUGUE produced an unwarping shift map that represented the magnitude of each voxel's shift from the original signal source in each EPI because of magnetic inhomogeneity. The unwarping direction depended on the phase encoding direction of EPI acquisition (posterior–anterior). Then, EPIs with and without DC and the unwarping shift maps were registered to structural images and the MNI (Montreal Neurological Institute) atlas using FNIRT (nonlinear registration with FMRIB's Nonlinear Image Registration Tool) and resampled to 4 × 4 × 4-mm resolution. Two datasets, one with distortion correction (DC+) and the other without correction (DC−), were concatenated across all subjects into a single 4D dataset for group ICA analysis.

### Component identification and selection

**Figure 2:**
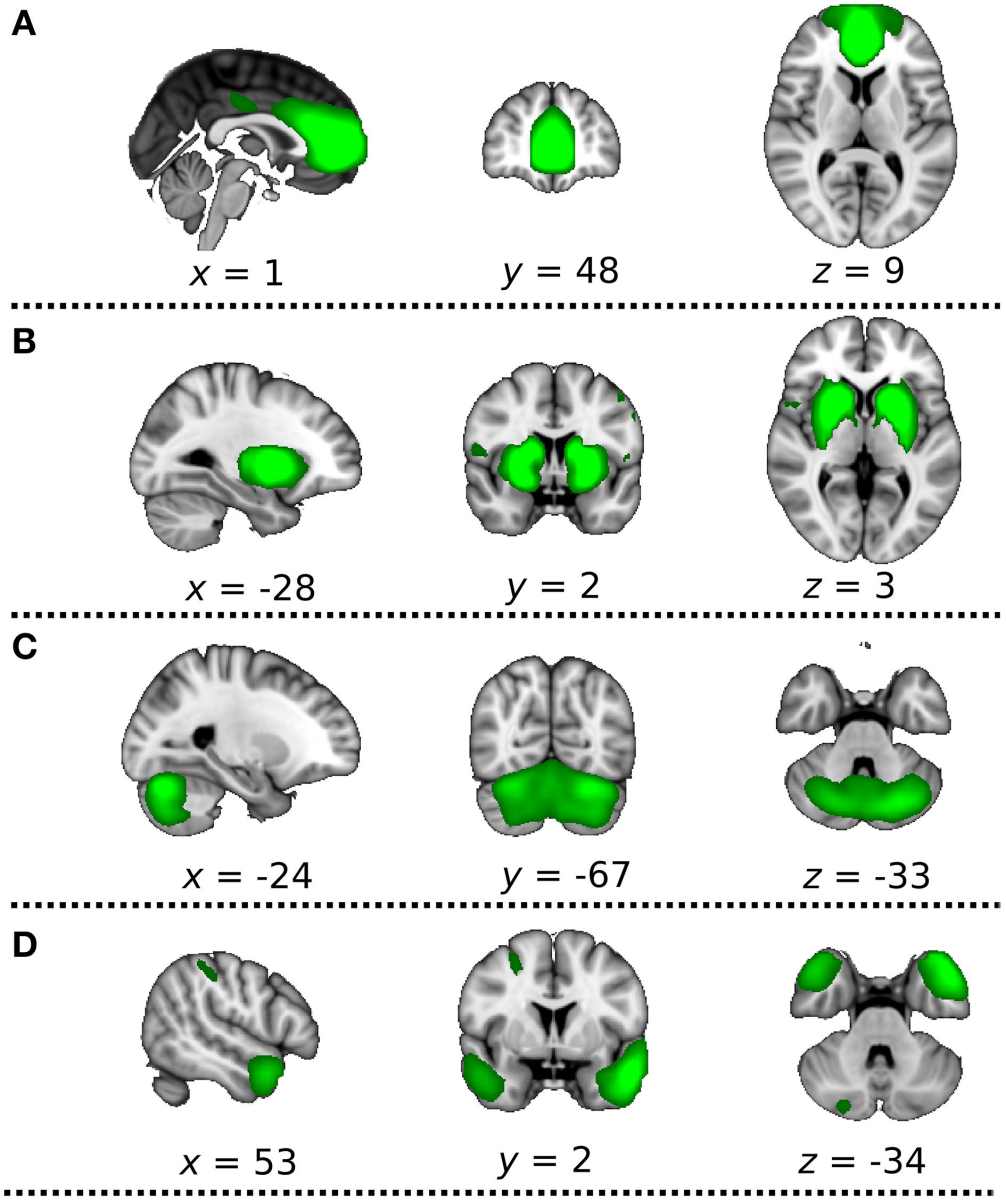
Four independent component analysis (ICA) components of interest. The most informative slices are shown. Green indicates extracted ICA networks, **(A)** anterior default mode network, **(B)** basal ganglia network, **(C)** cerebellum network, **(D)** temporal pole network. These networks are thresholded at approximate default threshold used by MELODIC for visualization purposes.

Group-spatial ICA was conducted on the rs-fMRI data including both DC+ and DC− versions to avoid a bias toward detecting resting state fluctuations characteristic of either the DC+ or DC− version. The estimation of the component dimension was performed automatically according to dimensionality estimation, resulting in 77 spatial components. Later, the components were identified visually (JR and HT) according to the Harvard-Oxford cortical atlas and subcortical structural atlases (Frazier et al., 2005; Desikan et al., 2006; Makris et al., 2006; Goldstein et al., 2007; Figure 2). Four of those were identified as the ICA components of interest [i.e., the anterior default mode network (DMN), basal ganglia network (BGN), cerebellum network (CBLN), and temporal pole network (TPN)] because these networks are near the paranasal sinuses or the mastoid air cells and vulnerable to distortion caused by magnetic inhomogeneity.

### Assessment of degrees of voxel shift after distortion correction

We evaluated the estimated degree of shift applied to the rs-fMRI voxels using the unwarping shift map derived from the field map images. We calculated the mean and standard deviation of the degree of shift (mm per voxel) in the representative cluster of each ICA of interest (Figure 2). The representative cluster was defined as the largest cluster of each ICA map approximately at the default threshold used by MELODIC.

### Assessment of spatial distribution of functional connectivity

All 77 of the spatial maps from the group ICA were used to generate subject-specific versions of the spatial maps and associated time series using the dual regression approach (Filippini et al., 2009). First, for each subject, each single spatial map is regressed (as a spatial regressor in a multiple variable regression) onto the subject's 4D space–time dataset. This procedure yielded subject-specific time series sorted into group-level spatial maps. Next, these time series were regressed (again, as temporal regressors in a multiple variable regression) onto the same 4D dataset, resulting in subject-specific spatial maps sorted into group-level ones. For comparison between the two groups, we used a paired *t*-test implemented in Permutation Analysis of Linear Models (PALM) tool (DC+ > DC− and DC− > DC+), which provided non-parametric family wise error (FWE) correction over multiple voxels, the 2 contrasts and the ICA masks of interest (Figure 2) simultaneously (Winkler et al., 2014, 2016). For statistical inference, we used a threshold *p* < 0.05 corrected for FWE using threshold-free cluster enhancement (TFCE) (Smith and Nichols, 2009). This approach is considered to be fairly conservative and strong against false positives. In the dual regression analysis, we also used a height-level threshold of *p* < 0.05 family-wise error (FWE) corrected for multiple comparisons to test the effects of different thresholding methods.

To assess the difference among methods to retrieve RSN, we also performed a seed-based correlation analysis focusing on the anterior DMN. The seed region corresponding to the anterior DMN was selected according to the group ICA map. For the statistical comparison between the DC+ and DC- datasets, we used a paired *t*-test implemented in the PALM. For the statistical inference, we used a height-level threshold of *p* < 0.05 FWE-corrected for multiple comparisons across the voxels and 2 contrasts (DC+ > DC− and DC− > DC+).

### Assessment of rs-fMRI signal quality

We examined how DC influenced the signal quality derived from resting-state BOLD signal fluctuations by finding the signal-to-noise ratio of the rs-fMRI time series. Specifically, we analyzed the BOLD signal spectrograms in the rs-fMRI time-series using Matlab 2013a (Mathworks Inc., USA). The temporal frequency of rs-fMRI signals of neuronal origin ranges 0.01–0.1 Hz, while the noise components of rs-fMRI time-series tend to show high-frequency components: for example, more than 50% of signal frequency components over 0.1 Hz is a criterion for rs-fMRI noise (Kelly et al., 2010). Thus, the ratio of low-frequency to high-frequency signal power (LFHF ratio) was considered to represent rs-fMRI's ability to detect BOLD fluctuations of neuronal origin over noise. The LFHF ratio was defined as the total power of rs-fMRI signals 0.01–0.1 Hz divided by the total power of rs-fMRI signals at frequencies >0.1 Hz (data acquisition was performed at 0.4 Hz). The LFHF ratio maps were calculated in both the DC+ and DC− datasets voxel-by-voxel. To evaluate the effects of DC on the LFHF ratio, we compared the LFHF ratio between the DC+ and DC− datasets in two ways. First, we computed the ratios of the voxel values in the DC+LFHF map to those in the DC−LFHF map for each participant, then averaged the maps over participants for qualitative analysis. Second, for statistical comparison between the two groups, we used a paired *t*-test implemented in the PALM tool that was similar to the comparison of functional connectivity. We performed non-parametric FWE correction across voxels and 2 contrasts (DC+ > DC− and DC− > DC+). For statistical inference, we used a significance threshold of *p* < 0.05 corrected for FWE using TFCE and a height-level threshold.

### Assessment of functional connectivity by synthetic data

With the empirical analysis above, there is no way of knowing the grand truth. To overcome this limitation, we produced 30 synthetic fMRI data sets according to each individual's real fMRI data. First, a dummy 4D rs-fMRI time-series (237 volumes) was created for each participant using the same number of copies of the mean EPI image derived of each individual's real fMRI data. Second, we created two spherical volumes-of-interest (VOIs) with a 10-mm radius centered at the MNI coordinate of *x* = 2, *y* = 42, *z* = 4 (corresponding to the anterior cingulate cortex, ACC) and at the MNI coordinate of *x* = 2, *y* = −46, *z* = 28 in (corresponding to the posterior cingulate cortex, PCC). Synthetic time-series was created for each data set, serving as the time series data in all voxels of both masks. In other words, the all the voxels in the ACC and PCC mask VOI had the identical signal time-series in each dummy dataset. Then, white noises were added to the time series data of each voxel to mimic the thermal noise of the real fMRI data time series. The amplitude of the white noises was adjusted so that the signal-to-noise ratio was equal to one (arbitrarily). Third, these synthetic datasets created in the MNI space were transformed into each individual's original space, and were distorted using the inverse of the matrix that had been produced from each participant's field map. Two copies of the distorted synthetic datasets were created: one underwent the DC procedure by FUGUE (DC+) and the other did not (DC−). The synthetic data with and without DC were both registered to structural images and the MNI atlas using FNIRT and resampled to 4 × 4 × 4-mm resolution. To identify spatial shift of VOIs in the DC+ and DC- datasets, group ICA analysis was conducted separately for each dataset. To assess the known functional connectivity, we performed a seed-based analysis, using the 10-mm radius spherical ACC VOI. For statistical comparison between the DC+ and DC- synthetic datasets, we used a paired *t*-test implemented in the PALM. We performed non-parametric FWE correction across voxels and 2 contrasts (DC+ > DC− and DC− > DC+). For statistical inference, we used a significance threshold of *p* < 0.05 corrected for FWE using peak-based thresholding method.

## Results

### Degrees of voxel shift after distortion correction

**Figure 3:**
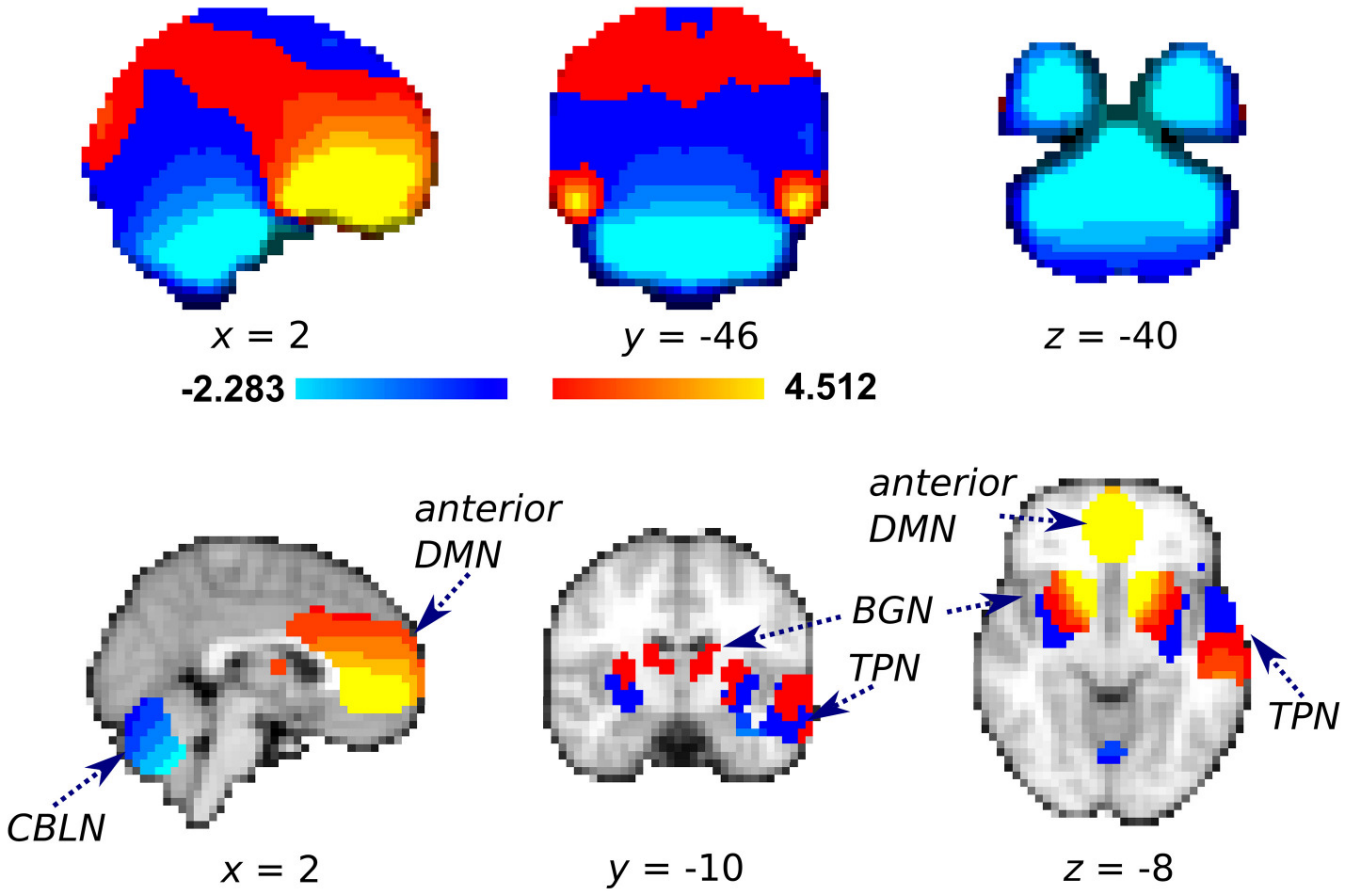
Averaged unwarping shift map of the whole brain **(Top)** used for field map correction and the same information restricted to the representative cluster of each independent component analysis of interest **(bottom)**. Red-yellow color: voxels were shifted anterior–posterior (mm per voxel); Blue-light blue: voxels were shifted posterior–anterior. After successful distortion correction, voxels with positive and negative values should be shifted anterior–posterior and posterior–anterior, respectively. DMN, Default mode network; BGN, basal ganglia network; CBLN, cerebellum network; TPN, temporal pole network.

We first evaluated the estimated degree of shift applied to the rs-fMRI voxels using the unwarping shift map derived from the field map images. Almost all voxels in a representative anterior DMN cluster were shifted anterior–posterior (2.7 ± 1.6 mm, mean shift ± standard deviation) (Figure 3). Conversely, the voxels in a representative CBLN cluster were uniformly shifted in the posterior–anterior direction (−2.4 ± 1.2 mm). In contrast to the homogenous shift map within the DMN and CBLN, the shift direction of the BGN and TPN voxels depended on location within each cluster (mean shift in BGN: 1.3 ± 1.5 mm; mean shift in TPN: −1.3 ± 1.8 mm). Particularly, the voxels in the anterior sector of the BGN were shifted anterior–posterior.

### Functional connectivity analysis

**Figure 4:**
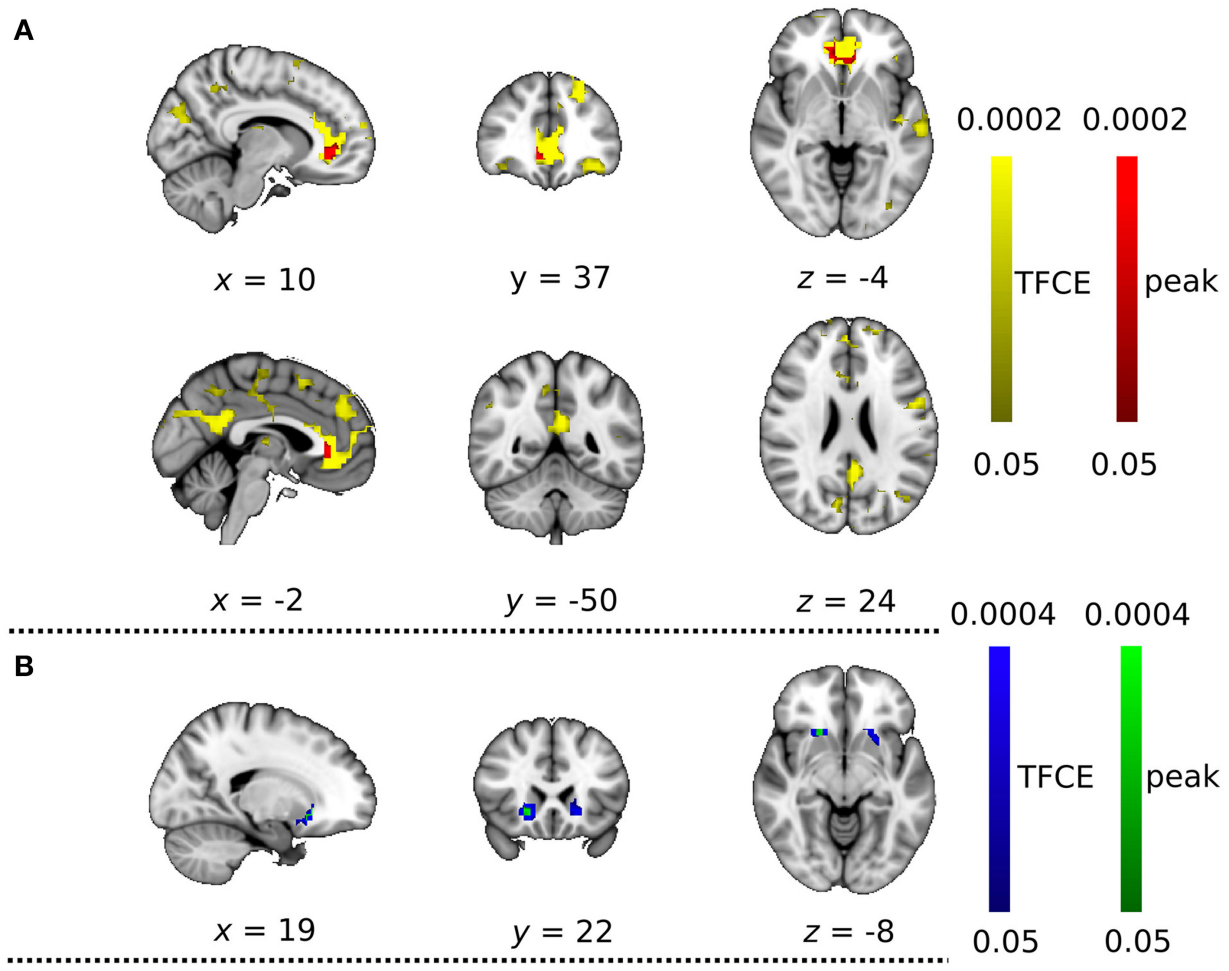
Functional connectivity with field map correction greater than that without correction as revealed by the dual regression analysis. **(A)** Anterior default mode network and **(B)** basal ganglia network. Colored clusters indicate significantly increased connectivity after field map correction thresholded by threshold-free cluster enhancement (TFCE) (YELLOW or BLUE) and thresholded by peak (height)-based method (RED or GREEN) (both after multiple comparison familywise error correction across voxels, components of interest, and contrast; *p* < 0.05).

With the dual regression approach, we compared the strength of intra- and extra-network connectivity of the four RSNs between the DC+ and DC− datasets. Analysis of the anterior DMN showed significantly more connectivity in the DC+ than the DC− dataset in both the peak-level and TFCE thresholding methods (Figure 4, Table 1). Areas with increased functional connectivity with the anterior DMN after DC included the ACC, which are important nodes of the DMN, in the height-level FWE corrected threshold (corresponding to voxel-level *p* = 0.0002). The CBLN's areas of increased functional connectivity after DC were found within the CBLN itself (i.e., intra-network) with the TFCE (cluster-level *p* = 0.012), but this increased functional connectivity was not found in the peak height-level thresholding method (voxel-level *p* = 0.089). At first glance, DC in the BGN seemed to result in significantly decreased connectivity in the anterior BGN in the height-level threshold (voxel-level *p* = 0.0004) (Figure 4, Table 1). However, detailed observation revealed that most voxels with decreased functional connectivity in the DC+ dataset were found in white matter, extending only slightly into the anterior border of the basal ganglia (ventral caudate nucleus and nucleus accumbens). Hence, this finding mostly resulted from mislocalization of the BGN to white matter in the DC− dataset. No difference in functional connectivity was found in the TPN.

**Table 1:** List of clusters with significantly changed functional connectivity with field map correction in the peak level threshold method (compared with no correction; multiple comparison family wise error correction across voxels, components of interest, and contrast; *p* < 0.05).

**Cluster size**	**Significance**	**MNI coordinates of min *p*-value**	**Region**	**Left/Right**
**DMN: significant increased functional connectivity with field map correction, compared to without correction**				
47	0.0002	10, 38, −8	Anterior Cingulate Cortex, Paracingulate Gyrus, Frontal Medial Cortex	Right
**BGN: significant decreased functional connectivity with field map correction, compared to without correction**				
8	0.0004	18, 18, −12	Cerebral White Matter, Putamen	Right

**Figure 5:**
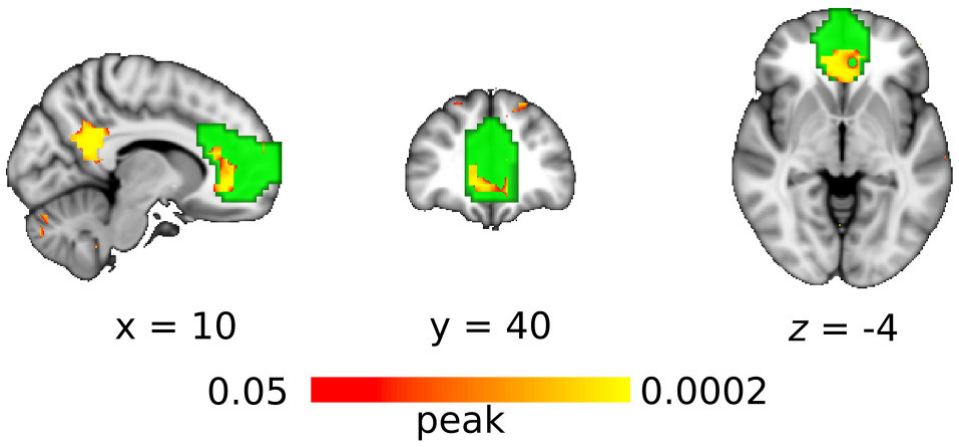
Functional connectivity with the DC (compared without DC) in anterior default mode network as revealed by the seed-based approach. Red-yellow indicates the clusters of significantly increased connectivity after DC (multiple comparison familywise error correction across voxels and contrast; *p* < 0.05). Green color indicates the region of interest in anterior default mode network.

With the seed-based analysis, the region of interest in anterior DMN showed significantly greater connectivity in the DC+ than the DC− dataset (Figure 5). Areas with increased functional connectivity were similar to the ICA-based approach and included ACC and PCC.

### Low frequency–high frequency ratio

**Figure 6:**
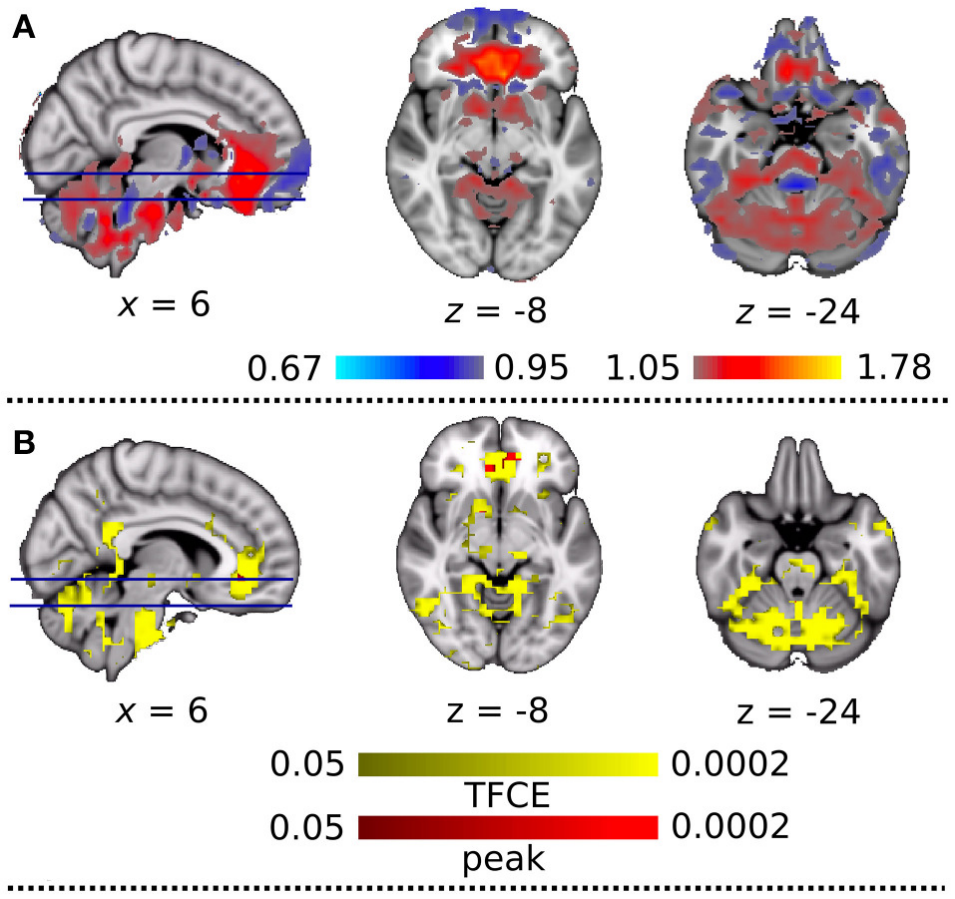
**(A)** Qualitative comparison map of the ratio of low-frequency to high-frequency signal power (LFHF ratio) between the datasets with and without field map correction. Red and blue indicate greater and lesser LFHF ratio after the correction, respectively. **(B)** Statistical comparison between LFHF ratio with and without field map correction. Colored clusters indicate significantly increased connectivity after field map correction thresholded by threshold-free cluster enhancement (TFCE) (YELLOW) and thresholded by peak (height)-based method (RED) (both after multiple comparison familywise error correction across voxels and contrast; *p* < 0.05).

To explain the above finding, we computed the LFHF ratio map from the viewpoint of DC+ and DC− rs-fMRI information quality (Figure 6A). Compared with the DC− dataset, the DC+ dataset showed a greater LFHF ratio in the ACC and cerebellum. Some brain regions showed a lower LFHF ratio in the DC+ than the DC− data, but those areas corresponded to white matter or the edge of the brain/structure. Detailed visual inspection revealed that imperfect spatial normalization of the DC− dataset caused this finding, which agreed with the mislocalization of the anterior BGN into the white matter without DC. Figure 6B shows a statistical comparison of the LFHF ratio between the DC+ and DC− datasets. The ACC showed significantly higher LFHF ratio in the DC+ dataset than the DC− dataset in the height-level threshold (*p* = 0.0002). Only a few voxels in white matter near the precentral gyrus showed significantly lower LFHF ratio in the DC+ dataset than the DC− dataset.

### Analysis of the synthetic data

**Figure 7:**
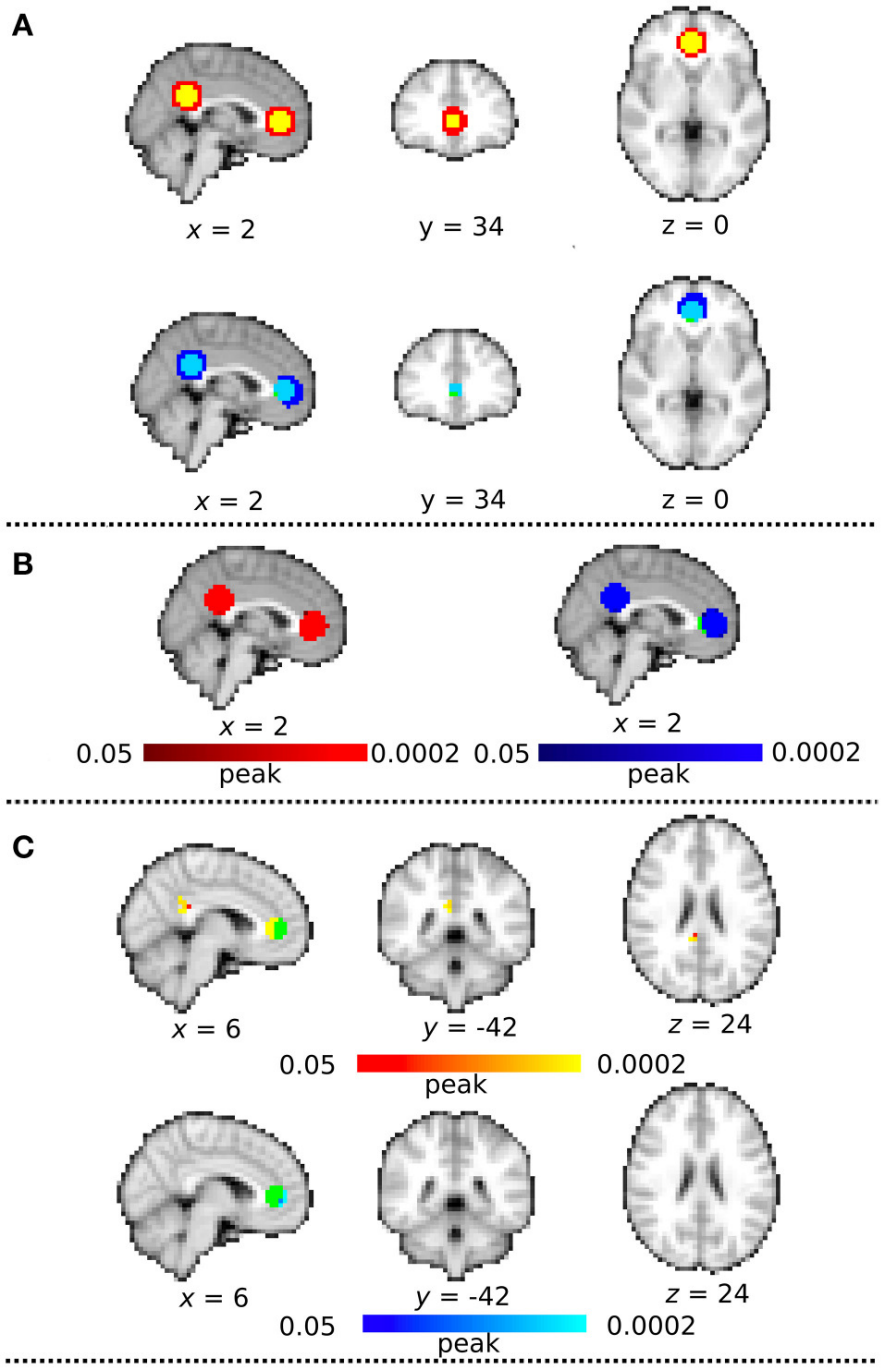
Synthetic MRI analysis. **(A)** The spatial correspondence between the masks and each group independent components analysis (ICA) map with distortion correction (DC+) and without DC (DC−). All clusters are binarized. Green color indicates the sphere mask image of 10-mm radius centered at the MNI coordinate of x = 2, y = 42, z = 4 (corresponding to ACC) and x = 2, y = −46, z = 28 (corresponding to PCC). Red indicates DC+ group ICA map and Blue indicates DC − group ICA map. Yellow indicates the region overwrapped between the masks and DC+ group ICA map, and lightblue indicates the region overwrapped between the masks and DC− group ICA map. **(B)** Group mean map in dataset with distortion correction (DC) (Red) and without DC (Blue). Green color indicates the sphere mask image of ACC and PCC volumes-of-interest (VOIs) (multiple comparison family wise error correction across voxels and contrast; *p* < 0.05). **(C)** Functional connectivity on the synthetic DC+ and DC− dataset. These clusters are masked by the ACC and PCC mask image to be focused on the voxels overwrapped between the masks and the functional connectivity. Green color indicates the region of interest in ACC. Red-yellow color indicates clusters of significantly increased connectivity after field map correction in peak-based thresholds (multiple comparison familywise error correction across voxels and contrast; *p* < 0.05). Blue-lightblue color indicates clusters of significantly decreased connectivity with field map correction in peak-based thresholds.

We evaluated the functional connectivity of the synthetic data which had the identical signal time-series in the spherical VOI in ACC and PCC. Figure 7A showed the spatial similarity between the masks and the ICA map derived from the DC+ and DC− datasets. The DC+ group ICA map covered all voxels in both the ACC and PCC VOIs. However, the DC− group ICA map was shifted posterior to anterior and did not cover a few voxels in ACC and PCC. The distance was extremely greater between centers of gravity of the ACC VOI and anterior part of DC- group ICA, corresponding to ACC (4.4 mm) than those of the ACC VOI and anterior part of DC+ group ICA, corresponding to ACC (0.49 mm).

Seed-based analysis showed significantly greater connectivity in the posterior part of the ACC mask (*p* = 0.0002) and also a part of PCC mask (*p* = 0.0002) in the DC+ than the DC− dataset (Figures 7B,C). The functional connectivity in DC- showed significantly greater connectivity at the anterior edge of ACC mask (*p* = 0.0002), but none in PCC mask (Figure 7C). These results supported superiority of localizing functional connectivity in the DC+ dataset over the DC- dataset.

## Discussion

This study investigated the effects of DC with field map on functional connectivity analysis in rs-fMRI. We performed group ICA using the DC+ and DC− datasets, generating an unbiased set of RSNs. We then focused on several RSNs, including areas susceptible to EPI distortion (i.e., the DMN, CBLN, BGN, and TPN). DMN showed higher functional connectivity in the DC+ dataset than in the DC− dataset. Some decreases in functional connectivity were noted in the BGN, primarily in white matter, indicating imperfect registration and normalization of the DC− dataset. These results were replicated with two different thresholding methods, the peak height-level thresholding and the TFCE. Moreover, supplementary seed-based and simulation analyses supported the results of the ICA-based functional connectivity analysis. We found a higher LFHF ratio after field map correction in the ACC, PCC, ventral striatum, and cerebellum. Overall, the results supported our hypothesis that the DC procedure increases the detectability of intra- and extra-network RSN connectivity, especially in regions susceptible to magnetic inhomogeneity. In task-fMRI studies, field map correction reduces variation in activated regions during motor or auditory tasks across subjects and improves statistical power (Cusack et al., 2003). To our knowledge, the present study is the first to show that DC improves the quality of findings in rs-fMRI analysis. Particularly, we demonstrated that detection of the RSN prototype (i.e., the DMN) was strongly affected by DC.

Consistent with a previous study (Jezzard and Balaban, 1995), the present shift map indicated strong effects of magnetic field inhomogeneity in the ventral/medial prefrontal areas/ACC, corresponding to the anterior DMN and cerebellum. In these areas, voxels were shifted by 75–85% of the original voxel size. Additionally, almost all voxels in the anterior DMN and cerebellum were shifted in the same direction: anterior–posterior in the anterior DMN and posterior–anterior in the cerebellum. As predicted, rs-fMRI analysis revealed more robust the functional connectivity in the DMN and CBLN in the DC+ dataset than in the DC− dataset, although the increased functional connectivity in CBLN was modest. These results contrasted with the findings in the TPN and BGN, in which no or only minor differences were found in functional connectivity between the datasets, as the direction of shift was more coherent in the anterior DMN and CBLN than in the TPN and BGN (e.g., Figure 3).

DC improved RSN detection in regions vulnerable to susceptibility. To determine how DC changed the quality of rs-fMRI data, we examined how it influenced the quality of BOLD signals that carry information relevant to rs-fMRI analysis. To achieve this goal, we defined the LFHF ratio across the whole brain and compared it between the DC+ and DC− datasets. We employed this approach because low-frequency BOLD signal fluctuations have been used to distinguish RSN from artifact-related “pseudo-networks” (Robinson et al., 2009). In essence, fMRI analyzes signals in the gray matter wherever synaptic/neuronal activity correlates with BOLD signals (Logothetis et al., 2001), depending on the levels of oxygenation and cerebral blood volume/flow (collectively called “hemodynamic responses”). This neurovascular coupling has been established in gray matter but not white matter (Rostrup et al., 2000; Preibisch and Haase, 2001). Conventional rs-fMRI studies typically target functional connectivity below 0.1 Hz (Biswal et al., 1995; Fransson, 2005; Fox et al., 2007). Moreover, low-frequency (0.01–0.1 Hz) BOLD signals contribute more than 90% of functional connectivity across regions, whereas high-frequency signals above 0.1 Hz reflect contributions from blood vessels, cerebrospinal fluid, and other physiological noise components (Cordes et al., 2001). Other studies also support the utility of the LFHF ratio as a marker of quality in rs-fMRI data: low-frequency fluctuations in white matter are reduced relative to those in gray matter by about 60% (Biswal et al., 1995). The signal's power spectrum within the CSF space is dominated by high-frequency fluctuations (Kelly et al., 2010), and the power spectrum of the rs-fMRI time series in the suprasellar cistern is higher than that of the PCC, especially in the higher-frequency range (Zou et al., 2008). In sum, the power spectrum of rs-fMRI time series in white matter and CSF is dominated by high-frequency fluctuations (>0.1 Hz), and that in gray matter is dominated by low-frequency fluctuations (0.01–0.1 Hz). Thus, the LFHF ratio reasonably represents the quality of rs-fMRI data for RSN detection. We found that areas with increased RSN detectability nicely corresponded to the areas with increased LFHF ratios after DC, which likely improves detection of RSN functional connectivity, by aligning gray matter signals and reducing contamination with signals from white matter and CSF.

These findings should facilitate improvement of registration and spatial normalization after DC, affecting the localization of gray matter, white matter, CSF, and the borders between these structures across participants (Hutton, 2002). For example, improvement of the anterior DMN signal could result from more precise registration of the ventromedial prefrontal cortex/ACC, anterior lateral ventricle proximal to the ACC, and the surrounding white matter, which were moved anterior–posterior to match them to the structural image. The improvement of registration after DC probably enhanced the detection of resting state functional connectivity of the anterior DMN with the ACC. We hypothesized that BGN would show greater functional connectivity with than without field map correction because the anterior parts of the ventral striatum are susceptible to inhomogeneity. Unexpectedly, however, the BGN analysis seemingly revealed the opposite results. The areas with decreased connectivity with the BGN corresponded mainly to white matter, extending only slightly into the anteromost part of the ventral striatum. The BGN voxels with decreased functional connectivity tended to show lower LFHF ratio in the data with than without DC, although the difference did not reach statistical significance. This indicates incorrect assignment of BGN voxels to white matter in the standard template because of imperfect normalization of the DC− dataset. In other words, the anterior parts of the ventral striatum were mislocalized to white matter in the DC− dataset, thereby producing “pseudo intra-connectivity”; as these voxels were shifted posteriorly to the correct location in the DC− dataset, the pseudo-connectivity disappeared and was correctly replaced by white matter signals.

A limitation of this study was that we acquired EPI rs-fMRI images in the posterior–anterior phase encoding direction only. The difference in phase encoding direction affects EPI distortion, but we concentrate on assessing the rs-fMRI protocol that is now widely used in Japanese cohort studies. Another limitation was that we only tested DC with field map. A recent study reported an alternative DC method that acquires EPIs in opposite phase encoding directions, resulting in opposite spatial distortion patterns, from which the unwarping field is computed (Andersson et al., 2003; Holland et al., 2010). In future studies, we should compare the effects of different phase encoding directions and DC methods on RSN detectability.

In summary, we performed ICA-based functional connectivity analysis of rs-fMRI data with and without field map DC, which improved statistical power for detection of functional connectivity analysis in the ACC associated with the DMN. In addition, these finding were supported by seed-based functional connectivity analysis and the simulation analysis. We did not find any significant drawbacks in the analysis with DC. We found a higher LFHF ratio in the ACC, PCC, ventral striatum, and cerebellum after DC, suggesting that improvement in signal assignment to anatomical segments improves the analysis. We suggest that researchers should include a field map DC step in the preprocessing pipeline, especially when they are interested in functional connectivity in the DMN.

## Author contributions

Guarantors of integrity of entire study: THa; study concepts and study design: HT and THa; data acquisition: THi and HM; data analysis and statistical processing: HT, JR, and KY; interpretation of data for study: HT, JR, KY, NH, and THa, drafting of manuscript: HT and JR; supervising manuscript: THa, manuscript final version approval: HT, JR, KY, THi, HM, NH, and THa.

### Conflict of interest statement

The authors declare that the research was conducted in the absence of any commercial or financial relationships that could be construed as a potential conflict of interest.
